# Instability Prevention and Treatment in Total Hip Replacement

**DOI:** 10.1055/s-0045-1810122

**Published:** 2025-12-10

**Authors:** Bruno Alves Rudelli, Fabio Seiji Mazzi Yamaguchi, Marco Rudelli, Lucas Torres Oliveira, Helder de Souza Miyahara, Henrique Melo de Campos Gurgel

**Affiliations:** 1 Institute of Orthopedics and Traumatology, Hospital das Clínicas da Faculdade de Medicina da Universidade de São Paulo (IOT-HCFMUSP), São Paulo, SP, Brazil; 2 Department of Orthopedics and Traumatology, Santa Casa de Misericórdia de São Paulo, São Paulo, SP, Brazil

**Keywords:** arthroplasty, replacement, hip, joint dislocations/prevention & control, joint instability, postoperative complications, artroplastia de quadril, complicações pós-operatórias, instabilidade articular, luxações articulares/prevenção & controle

## Abstract

Instability is a challenging complication and a significant revision cause in hip replacement surgery. The incidence of dislocation ranges from 0.5 to 10% in primary prostheses and can reach up to 30% in revision surgeries. The literature describes several risk factors, including surgeon-, patient-, and implant-related factors. Knowledge of these factors is essential to preventing and managing instability cases. Preventive treatment involves preoperative planning, adequate component positioning, normal hip biomechanics reestablishment, risk factor identification, and proper implant selection. Approximately two thirds of dislocation episodes are treatable with non-surgical treatment with closed reduction, education, and muscle strengthening. One third of the cases develop recurrent dislocations and require surgical intervention. Revision surgery should target the cause of instability. When necessary, consider special implants, such as dual-mobility acetabular components, polyethylene-based lipped acetabular liners, constrained acetabular inserts, or large-diameter prosthetic heads. Technological advances in robotic surgery and the understanding of the biomechanics of spinopelvic swing-related dislocation are promising current topics that may improve the prevention and treatment of instability.

## Resumo

A instabilidade é uma complicação desafiadora e uma das principais causas de revisão na cirurgia de prótese de quadril. A incidência da luxação varia de 0,5 a 10% nas próteses primárias e pode chegar a 30% nas cirurgias de revisão. Existem diversos fatores de risco descritos na literatura, sendo que podemos dividi-los em dependentes do cirurgião, dependentes do paciente e dependentes do implante. O conhecimento pleno desses fatores é fundamental para a prevenção e manejo da instabilidade. O tratamento preventivo envolve planejamento pré-operatório, posicionamento adequado dos componentes, restabelecimento da biomecânica normal do quadril, identificação de fatores de risco e escolha adequada do implante. Aproximadamente dois terços dos episódios de luxação podem ser tratados não cirurgicamente com redução fechada, orientações e fortalecimento muscular. Um terço evoluiu para luxações recorrentes e necessita de intervenção cirúrgica. A cirurgia de revisão deve ser direcionada diretamente para a causa da instabilidade. Quando necessário, considerar a utilização de implantes especiais como acetábulos de dupla mobilidade, insertos de polietileno com rebordo elevado, acetábulos constritos ou cabeças de grande diâmetro. Avanços tecnológicos na área da cirurgia robótica e na compreensão da biomecânica da luxação relacionada ao balanço espinopélvico são temas atuais promissores que devem impactar em melhorias na prevenção e no tratamento da instabilidade.

## Introduction

Instability in total hip arthroplasty is a significant and stressful complication and one of the main current causes of surgical revision. 1 Although there is no Brazilian national data registry, information from countries with such databases shows that instability is a critical revision cause. In the Australian registry, instability is the third most common cause for revision, accounting for 14.6% of cases; 2 in the Danish registry, it is the second cause, with a 17% rate; 3 and in the United States, instability is the main indication for revision, with a 17.3% rate. 4

The incidence of dislocation after primary hip arthroplasty varies considerably in the literature, depending on the population studied, ranging from 0.5 to 10%. 5 In revision arthroplasty, this incidence can reach up to 30%. 6 Historically, in the 1st case series, Charnley reported a dislocation incidence of 4.8%. 7 However, this number has been decreasing over time. In a meta-analysis by van Erp et al. 8 on the incidence of dislocation from 1960 to 2020, the average dislocation rate after total hip arthroplasty was 3.7% from 1960 to 1970 and decreased to 0.7% from 2010 to 2020. This reduction likely reflects technical and conceptual advances in the management and prevention of instability.

In most case series, 60 to 70% of dislocations occur within the 1st 6 weeks after surgery. 9 After this period, the estimated cumulative risk of dislocation is 1% every 5 years from the initial surgery. 7 Late dislocations may occur due to predisposing factors with delayed manifestation, such as improper component positioning, or to new risk factors, including polyethylene wear and neurological conditions. 7

## Risk Factors

The literature describes several risk factors for instability, some with more robust statistical support and others with a certain degree of questioning regarding their actual association with dislocation episodes. In practical terms, there are three risk factor categories for instability: patient-related factors, surgeon-related factors, and implant-related factors.

### Patient-Related Factors

#### Gender

Although the risk of instability in female patients was always deemed higher, 10 more recent systematic reviews and data from national registries have shown no direct association between dislocation and gender. 1 These discrepancies in the literature probably result from confounding biases, including the increased association of females with conditions such as developmental hip dysplasia, femoral neck fractures, and their smaller acetabular sizes, which preclude the placement of larger heads.

#### Age

Several studies reported a higher risk of dislocation in older patients, although there is no consensus on the specific age range associated with this risk. 11 Miscellaneous aging-related conditions, including loss of lumbosacral spine mobility, sarcopenia, increased fall risk, and neurological diseases, may explain these findings. Interestingly, some recent studies suggested a potential increased risk of dislocation in younger patients, especially those under 50. 12 The greater functional demand and the potential presence of more complex joint conditions in this population may justify this phenomenon.

#### Neuromuscular Diseases and Abductor Muscle Failure

A myriad of conditions, due to different mechanisms, can increase the likelihood of dislocation resulting from abductor muscle failure, muscle contractures, loss of lumbosacral spine mobility, and lack of cooperation with movements posing a risk for dislocation. These illnesses include Parkinson's disease, cerebral palsy, multiple sclerosis, poliomyelitis sequelae, stroke sequelae, and spinal cord injuries, and can increase the dislocation risk to 7.5 to 10.6% of cases in some series. 13 Abductor muscle failure may occur in other situations unrelated to neuromuscular diseases that increase the instability risk, such as revision surgeries with pseudoarthrosis or massive greater trochanter osteolysis, disarthrodesis, and extensive tendon attachment loss to the gluteus medius. 14

#### Obesity

Obesity, defined as a body mass index (BMI) above 30 kg/m ^2^ , has been associated with an increased risk of dislocation and may present a 2.5-fold risk ratio compared with the non-obese population. 15 However, some more recent studies have cast doubt on this issue as they found no association between BMI and dislocation episodes. 5 11 A potential reason for the higher dislocation risk in obese patients is inadequate component positioning due to surgical approach-related technical difficulties resulting from the copious amount of subcutaneous tissue. However, to date, this correlation remains statistically insignificant. 1 Interestingly, a retrospective cohort of more than 150 thousand patients presented data suggesting a higher dislocation risk in patients with a BMI lower than 20 kg/m ^2^ . 10 Dislocation studies rarely evaluate patients with low BMIs separately, and this may be an underestimated factor in the literature.

#### Spinopelvic Swing

A little over a decade ago, the concept of spinopelvic swing, including the relationship between lumbosacral spine mobility and the spatial positioning of the acetabulum, emerged as another determining factor in postarthroplasty hip stability. 16 Patients with a rigid system resulting from lumbosacral spine arthrodesis or ankylosis, such as in ankylosing spondylitis, can present a considerable risk increase. 17 An interesting study by Buckland et al. 18 comparing patients who underwent total hip replacement without lumbar spine conditions and those who underwent arthrodesis analyzed the number of instrumented levels and revealed that the greater the number of levels, the greater the risk of dislocation. The relative risk can be 2.7 times for 3 or more fixed levels. Although it remains uncertain how and to what extent this rigidity of the spinopelvic swing influences the usual positioning of the total hip prosthesis, 19 this topic represents a promising field for future scientific advances.

#### Specific Conditions

Some conditions may increase the risk of dislocation, including femoral head osteonecrosis and rheumatological diseases. 15 According to Yang et al., 20 the risk of an episode of dislocation occurring 1 year after total hip replacement surgery performed for osteonecrosis is 1.48 times higher than in patients with primary osteoarthritis. This data is consistent with another more recent systematic review and meta-analysis reporting a 1.5 times higher relative risk for osteonecrosis cases. 21 Although developmental hip dysplasia is a complex subject due to its diverse clinical presentations, technical challenges, and different bone reconstruction approaches, most recent evidence showed no association with a higher risk of instability. 22 Nevertheless, Komiyama et al. 23 documented a higher risk of dislocation when acetabular reconstruction occurs 2.39 cm or more above its physiological center.

Another important risk factor is arthroplasties for femoral neck fracture treatment, which can present a 10-fold higher risk of dislocation when compared to elective surgeries. 11 13 Patients with femoral neck fractures often are frail elderly subjects with many of the risk factors mentioned previously, including advanced age, neurological/cognitive abnormalities, abductor muscle weakness, and lumbosacral spine rigidity. For these cases, surgeons commonly select implants with a lower risk of dislocation, such as constrained or dual-mobility prostheses, or proceed to hemiarthroplasties.

### Surgeon-Related Factors

#### Surgical Approach

Historically, the posterolateral approach has been associated with an increased risk of dislocation, probably because it is the only classic approach that exposes the joint through a posterior capsule opening. However, more recent studies evaluating the risk of dislocation in the posterior approach with capsular repair showed a similar incidence of instability. 24 A systematic review by Kwon et al. 25 reported that the relative risk of dislocation in the posterolateral approach with no repair could be up to 8.2 times higher depending on the case series evaluated. However, a comparison of the posterolateral approach with repair to the direct anterior approach and the lateral approach revealed no statistically significant difference. Recent meta-analyses evaluating different surgical approaches corroborated this conclusion. 26 The current trend is to implement and develop approaches that increase the sparing of muscles and tendons, such as the direct anterior approach and, more recently, the Supercapsular Percutaneously-Assisted Total Hip (SuperPATH) and Save the Piriformis And obturator Internus, Repair Externus (SPAIRE) approaches. 27 Nevertheless, no relevant evidence demonstrated superior stability after these approaches. 28

#### Component Positioning

The most widely used parameter for component positioning is the classic safety zone described by Lewinnek, with respective acetabular inclination and anteversion values of 40 ± 10° and 15 ± 10°. 29 However, these parameters received many criticisms because the original work had several biases, and many subsequent studies were unable to reproduce Lewinnek's findings with statistical significance. 30 Another famous concept is the combined anteversion presented at the Ranawat test, described in 1994 31 and consisting of the sum of femoral and acetabular versions. In a broad study using navigation, Dorr et al. 32 defined a safety zone of combined anteversion ranging from 25 to 50°. As dislocations still occur even when respecting all these parameters, some authors stated that it is not possible to define a safety zone for component positioning. 5 Instability is multifactorial, and it is often difficult to effectively isolate the various risk factors that can lead to dislocation, including individual anatomical variations. In the future, technological advances, including three-dimensional tomography and biomechanical analyses considering sagittal balance and individual variations, may define the ideal positioning for each patient.

#### Anatomy Reestablishment

Reconstructing the physiological and anatomical parameters of hip offset, length, and center of rotation is fundamental for proper biomechanical prosthetic function, as it prevents impingements between the prosthetic components and bone structures that cause dislocation from happening. This reconstruction requires meticulous surgical planning and selection of implants that allow the restoration of the patient's physiological anatomy. The current transition from printed radiograph-based planning to digital planning has been causing several adaptation difficulties. However, as technology improves, digital planning accuracy increases. 33

### Implant-Related Factors

#### Femoral Head Size

The femoral head size is a subject widely discussed in the literature since the head diameter influences the range of motion and jumping distance. 34 Most studies showed that femoral heads smaller than or equal to 28 mm are risk factors for dislocation, while heads greater than or equal to 32 mm are protective factors. 35 However, it is controversial whether a real benefit would derive from using heads larger than 36 mm due to polyethylene volumetric wear, risk of acetabular insert fracture, head impingement on soft tissues causing inguinal pain, trunnionosis, and local adverse tissue reaction

#### Lipped Acetabular Liners

Polyethylene-based lipped acetabular liners may protect against dislocation by increasing the distance that the femoral head needs to travel to disengage from the acetabular component. 6 Although there is some concern about the possibility of the femoral component impinging on the lipped liner, resulting in a paradoxical increase in the risk of dislocation and early loosening, systematic reviews and cohort studies have not shown complications associated with these implants. 36

#### Tribology

Polyethylene acetabular component wear may result in late dislocation, especially after 5 years of the initial surgery, 7 due to ligament laxity caused by the rise of the head in the worn liner and local microinstability. 13 Therefore, some authors have suggested the use of the ceramic/ceramic tribological pair as an alternative to avoid late dislocation. However, the advancement of crosslinked polyethylene reduced long-term wear, which no longer influences the cumulative risk according to national data records. 37

#### Constrained Acetabular Inserts

Constricted acetabular components are implants presenting a locking femoral head mechanism to the polyethylene insert. The literature presents considerable controversy regarding their use. Some series report excellent outcomes, with failure rates ranging from 1.8 to 2.4% in a mean follow-up time of 4 to 5 years, especially in patients at high risk of instability. 38 39 In contrast, other investigations suggested that these implants should be used as salvage procedures, presenting failure rates from 10 to 23%. 40 41

The main concern is the limited range of motion within physiological values, which may generate excessive stress at the implant/bone interface or between the femoral component and the locking mechanism. This phenomenon may result in premature loosening, accelerated polyethylene wear, or failure of the constriction mechanism leading to implant failure. 40 It is worth emphasizing the availability of implant designs with different locking mechanisms, each with different outcomes. 42 Implants with a lower range of motion restriction often present more positive outcomes, including tripolar implants 43 and those with recesses in the most common impingement areas to reduce motion restriction. 44

#### Dual-Mobility Acetabular Components

Dual-mobility prostheses are not new, as their development by Bousquet et al. 45 happened in France in 1973. However, due to complications from early polyethylene wear and the high incidence of intra-prosthetic dislocation using the first devices, 46 the literature on dual-mobility acetabular components gained widespread prominence only in 2009, after their approval by the United States Food and Drug Administration. 47 Technological advances in implant design, currently in its third generation, and the advent of crosslinked polyethylene reduced these complications substantially. A more recent meta-analysis evaluating elective primary prostheses demonstrates a survival rate for any cause of revision of 99.7% in 5 years and 96.1% in 15 years. 48 When compared with constrained acetabular components, the literature often favors dual-mobility components, as reported by another systematic review with meta-analysis by Donovan et al. 49 These authors showed a failure rate for dislocation of 3% for dual-mobility components versus 9% for constrained components, a revision rate for instability of 2% versus 9%, and a revision rate for any cause of 8% versus 19%. However, there is still concern about long-term survival and failure in special cases, such as severe abductor mechanism failure. 50

#### Navigation and Robotics

The introduction of surgical navigation in hip replacement arthroplasty occurred in 1998 by Jaramaz et al. 51 Surgical navigation consists of a system in which the patient's spatial position (based on intraoperative anatomical references provided by the surgeon) undergoes digital capture and processing by specialized software to provide real-time visual, graphical, or numerical information about the steps of the procedure, increasing component positioning control and precision. 52 Although the studies were promising regarding the precision of acetabular positioning, the literature has failed to demonstrate clinical outcome improvements. 53 Even though studies have shown greater precision in positioning components in the safety zone with robotic-assisted surgery (97%) when compared with navigation (84%) and manual methods (74%), 54 questions regarding the cost-benefit of this technique, considering the robot and operational cost, cast doubt on the advantage of systematically adopting this technique. 55 However, recent studies increasingly demonstrated that robotic assistance decreases the incidence of dislocation, especially in complex cases with spinopelvic swing dysfunction. 56 Robotic techniques have a promising future, particularly in the current scenario of artificial intelligence technology development.

## Prevention

Preventive treatment of instability requires technical optimization of all modifiable, that is, surgeon-related risk factors. The elements for consideration include:

- Detailed preoperative planning to accurately reconstruct the physiological positioning of the acetabular rotation center, global offset, and limb equalization.
- Surgical access respecting tissue integrity (regardless of the chosen approach), which requires special care in tendon and muscle manipulation, reattachment, and adequate capsular repair.
- Proper component positioning within the safety zones and combined anteversion.
- Identification of all patient-related risk factors to guide the selection of the implants potentially minimizing the risk of dislocation. Although these factors are not modifiable, options include implants with large-diameter heads, lipped acetabular liners, and dual-mobility or constrained components. Each implant has advantages and disadvantages requiring individual consideration.

## Non-Surgical Treatment

Approximately two thirds of dislocations may undergo non-surgical treatment with good outcomes, especially in those occurring within 3 months and presenting proper component positioning and anatomical reestablishment. 13 After closed reduction, patient guidance regarding movements that pose an instability risk and motor physical therapy for strengthening hip stabilizer muscles are essential. When there is no patient cooperation or significant muscle failure, hip abduction orthoses can be used for 8 to 12 weeks. Nevertheless, the literature shows scarce evidence to support the routine use of these devices. 57

## Surgical Treatment

Recurrent dislocations may require replacement revision. Although no defined number of dislocation episodes indicates surgical intervention, late dislocations or cases with poor component positioning have a higher association with recurrence and require a lower threshold for revision indication.

In these cases, the surgical approach targets instability cause; as a result, a careful analysis of all patient-related risk factors is essential, understanding that etiology is often multifactorial. To help with practical decision-making, Wera et al. 58 classified instabilities into six types ( Table 1 ) and Sheth et al. 59 presented guidelines for managing these instabilities.

**Table 1:** Etiological classification of instability 58

Type I	Inadequate acetabular component positioning
Type II	Inadequate femoral component positioning
Type III	Abductor muscle failure
Type IV	Bone or soft-tissue impingement
Type V	Asymmetrical polyethylene wear
Type VI	No identifiable cause

Type-I (acetabular component malpositioning) and II (femoral component malpositioning) instabilities must undergo revision of the compromised component to optimize anatomical parameter reestablishment. The evaluation of component positioning may use conventional radiographs in several views, but computed tomography leads to a more accurate understanding of positioning errors. 53

For type-III instability (abductor muscle failure), the indication is revision with constricted acetabular components. 59 The use of dual-mobility acetabular components in significant abductor mechanism failure has proven inconsistent. 50 If possible, abductor complex failure should be minimized by, for instance, repairing tendon lack of attachments to the gluteus medius and minimus and stabilizing greater trochanter pseudoarthrosis. Although the literature reports that muscle transfer techniques, allograft reconstructions, and distal trochanteric osteotomies improve functional patterns, it is unclear whether they have any impact on instability treatment. 14

In type-IV instability, dislocation occurs due to impingement resulting in a range of motion limitation potentially related to bone structures or the contracture of inadequate periarticular soft-tissue balance. 59 It is essential to identify the impingement etiology and remove it. Implants increasing the range of motion, such as large-diameter heads or dual-mobility acetabular components, are desirable in these cases.

Type-V instability results from asymmetric polyethylene insert wear, and it requires revision. If possible, use larger heads with highly cross-linked polyethylene to minimize instability recurrence. In fixed components not allowing a modular exchange of the polyethylene insert due to implant availability, cementation within a fixed metal cup has promising outcomes. 60

Type-VI instability features no apparent modifiable cause to justify the episodes of dislocation, and the suggested treatment is revision using constrained acetabular liners.

## General Considerations

Instability in total hip replacement is one of the main current complications, with a complex and multifactorial etiology. Understanding the risk factors, knowing the several implants available, and performing precise and individualized planning are essential for the prevention and treatment of this challenging complication.
